# Chemopreventive effects of atractylenolide II on mammary tumorigenesis via activating Nrf2-ARE pathway

**DOI:** 10.18632/oncotarget.20546

**Published:** 2017-08-24

**Authors:** Ting Wang, Fangyi Long, Xiqian Zhang, Yujie Yang, Xuehua Jiang, Ling Wang

**Affiliations:** ^1^ Department of Clinical Pharmacy and Pharmacy Administration, Key Laboratory of Drug Ministry of Education, West China School of Pharmacy, Sichuan University, Chengdu 610041, Sichuan, China; ^2^ Department of Pharmacy, Sichuan Cancer Hospital & Institution, Sichuan Cancer Center, School of Medicine, University of Electronic Science and Technology of China, Chengdu 610041, China; ^3^ Department of Pharmacy, Sichuan Provincial Hospital for Women and Children, Women and Children's Hospital of Chengdu Medical College, Chengdu Medical College, Chengdu 610041, China

**Keywords:** breast cancer, atractylenolide II, nuclear factor (erythroid-derived 2)-like 2, anti-oxidative response element, chemoprevention

## Abstract

In the studies of chemoprevention, the Nrf2-ARE signaling pathway has received widespread attention due to its anti-inflammatory and anti-oxidation effects. Our previous study indicated that atractylenolide II, which is an active component of *Atractylodes macrocephala Koidz*, is a potential activator of Nrf2-ARE signaling pathway. In this study, we observed that atractylenolide II significantly increased Nrf2 expressing, nuclear translocation and the expression of its downstream detoxifying enzymes, thus decreasing 17β-Estradiol induced malignant transformation in MCF 10A cells, and we found that atractylenolide II acted through JNK/ERK-Nrf2-ARE pathway. Furthermore, atractylenolide II significantly reduced N-Nitroso-N-methylurea induced tumor incidence, multiplicity and volume, with activation of Nrf2-ARE pathway and decreased inflammation and oxidative stress in rat mammary tissue. Collectively, our results suggested that atractylenolide II could protect against mammary tumorigenesis both *in vivo* and *in vitro* via activating Nrf2-ARE signaling pathway, which supported atractylenolide II as a novel chemopreventive agent of breast cancer.

## INTRODUCTION

Breast cancer is the malignant tumor arising from breast epithelial tissue, and it is the most common cause of death among women with cancer [1, 2]. Researches have shown that the carcinogenic progression could be effectively impeded or delayed by chemoprevention [3, 4], and several synthetic chemopreventive agents have been demonstrated to prevent the occurrence of breast cancer effectively (such as tamoxifen, raloxifene and exemestane), however, they were also reported to have intolerant adverse effects and low efficacy due to the single target mechanism [5–8], thus it is important to develop novel chemopreventive agents with high efficiency and low toxicity for breast cancer chemoprevention.

Emerging data has suggested that the incidence of breast cancer is associated with increased intracellular inflammation and oxidative stress, which is characterized by excess generation of reactive proinflammatory cytokines and reactive oxygen species (ROS) [9–12]. The nuclear factor E2-related factor 2 (Nrf2) is critically involved in the expression of the anti-inflammatory and antioxidant-associated enzymes, including NAD(P)H: quinone oxidoreductase 1 (NQO1) and heme oxygenase-1 (HO-1), which could protect against carcinogenesis [13–15].

**Figure 1:**
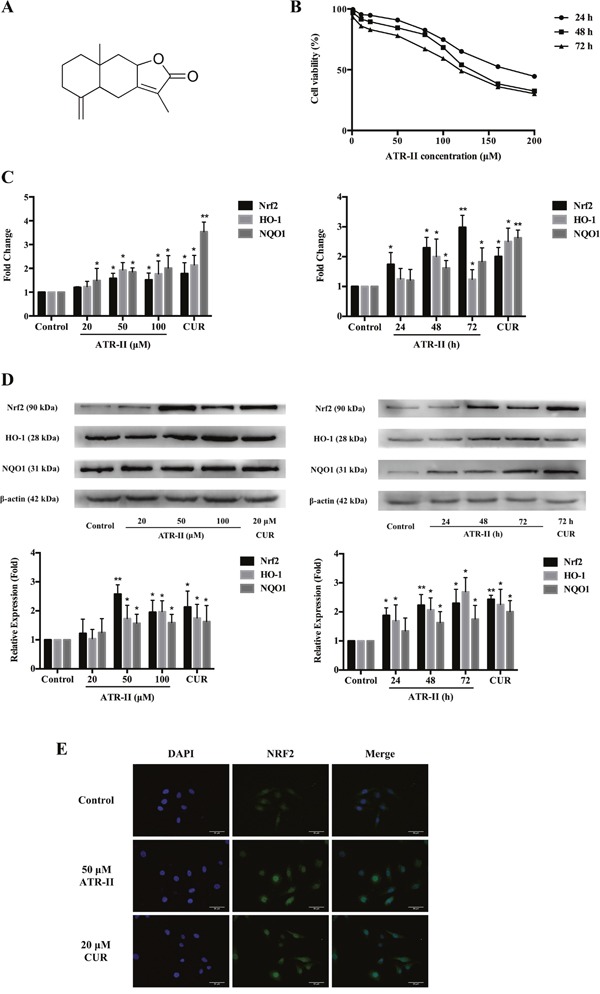
Effects of ATR-II on Nrf2 expression and nuclear accumulation in MCF 10A cells **(A)** Chemical structure of ATR-II. Its molecular weight is 232.32. **(B)** Cell viability of ATR-II in MCF 10A cells. Cells were treated with the indicated concentrations (1-200 μM) of ATR-II for 24, 48 and 72 h, respectively. Cell viability was determined using MTT assay. **(C)** Concentration- and time-dependent effects of ATR-II on the mRNA level of Nrf2 and it downstream gene (*HO-1* and *NQO1*) in MCF 10A cells. Cells were treated with the indicated concentrations (20, 50 and 100 μM) of ATR-II for 48 h, or treated with 50 μM of ATR-II for the indicated times (24, 48 and 72 h). **(D)** Concentration- and time-dependent effects of ATR-II on the protein expression of Nrf2 and it downstream gene (*HO-1* and *NQO1*) in MCF 10A cells. Cells were treated with the indicated concentrations (20, 50 and 100 μM) of ATR-II for 48 h, or treated with 50 μM of ATR-II for the indicated times (24, 48 and 72 h). **(E)** Nuclear translocation of Nrf2 induced by ATR-II (50 μM) for 48 h in MCF 10A cells, the cells were observed under epifluorescence microscopy. Scale bar, 50 μm. Significantly different (versus control group): *P<0.05 and **P<0.01.

In recent times, a large amount of naturally occurring anti-inflammatory and antioxidant agents are considered as promising chemopreventive agents. In our previous study, a cell based antioxidant response element (ARE)-driven luciferase reporter system was applied to screen potential Nrf2 activators among the compounds isolated from the rhizome of *Atractylodes macrocephala Koidz*, which is traditionally prescribed for breast cancer postoperative treatment by Chinese medicine practitioners, and the results suggested that atractylenolide II (ATR-II, Figure 1A) is a potential activator of the Nrf2-ARE signaling pathway. It was reported that atractylenolide had antioxidant and anti-inflammatory activities [16–18], thus ATR-II might be a candidate agent for breast cancer chemoprevention. However, the effects of ATR-II on breast cancer and the underlying mechanisms have not been elucidated.

In this study, we investigated the activation effects of ATR-II on Nrf2-ARE signaling pathway, and its anti-inflammatory, antioxidant and chemopreventive effects were further explored *in vitro* and *in vivo* using MCF 10A carcinogenesis model and rat breast cancer model, which for the first time reported the chemopreventive effects of ATR-II on breast cancer and its underlying mechanisms. In addition, the present study could provide further evidence for the molecular mechanisms that allow ATR-II to exert its potential role as a chemopreventive agent in mammary carcinogenesis.

## RESULTS

### ATR-II induced Nrf2 expression and nuclear accumulation in MCF 10A cells

Nrf2 is a key factor in protection against mammary carcinoma progression [19–21]. To investigate the effects of ATR-II on Nrf2-ARE signaling pathway activation, we examined the mRNA, protein levels and subcellur location of Nrf2 in MCF 10A cells after ATR-II treatment with the indicated concentrations and times, and CUR was chosen as a positive control which is widely accepted as an Nrf2 activator [15]. The cytotoxic effects of ATR-II were detected by MTT assay (Figure 1B). As shown in the Figure 1C and 1D, qPCR and Western blot analysis confirmed a significant increase of Nrf2 mRNA and protein levels after ATR-II treatment in dose- and time-dependent manners. Cell immunofluorescence analysis (Figure 1E) showed increased Nrf2 accumulation in the nucleus of MCF 10A cells after ATR-II treatment.

### ATR-II exhibited cytoprotective effects against LPS-induced inflammation and oxidative stress in MCF 10A cells

**Figure 2:**
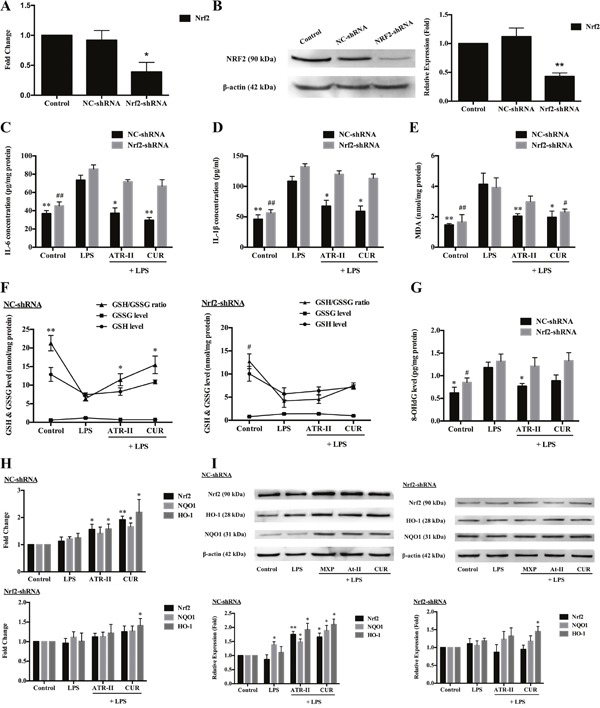
Protective effects of ATR-II on LPS-induced inflammation and oxidative stress in MCF 10A cells *Nrf2* knockdown attenuated the mRNA **(A)** and protein expression **(B)** levels of Nrf2 in MCF 10A cells. Pretreatment with ATR-II reduced the LPS-induced IL-6 **(C)**, IL-1β **(D)**, MDA **(E)** and 8-OHdG **(G)** levels, and increased the ratios of GSH/GSSG **(F)** in the NC-shRNA MCF 10A cells, while the *Nrf2* knockdown abolished these effects. ATR-II increased the mRNA **(H)** and protein expression **(I)** levels of Nrf2 and its downstream genes *NQO1* and *HO-1* in LPS-induced MCF 10A cells. Significantly different (A, B, H and I versus control group; C to G versus LPS group): *P<0.05 and **P<0.01 for NC-shRNA cells; #P<0.05 and ##P<0.01 for Nrf2-shRNA cells.

Previous studies have suggested that inflammation and oxidative stress played important roles in carcinogenesis [11, 22], and bacterial lipopolysaccharide (LPS) treatment has been widely used as a model of inflammation and oxidative stress [23, 24]. We have confirmed that ATR-II could upregulate Nrf2 expression, and Nrf2 is a candidate cytoprotective gene whose activation is associated with the increased anti-inflammatory and antioxidant gene expression, and the repression of colony formation in many cancer cell lines [25, 26], thus we continued to explore the protective effects of ATR-II against the LPS-induced inflammation and oxidative stress. In addition, to verify the functional role of Nrf2 in protecting against LPS-induced inflammation and oxidative stress in MCF 10A cells, NC-shRNA and Nrf2-shRNA MCF 10A cells were established using shRNA vectors. Deficient mRNA and protein levels of *Nrf2* were confirmed in Nrf2-shRNA cells by qPCR and Western blot analysis (Figure 2A and 2B). As shown in Figure 2C to 2G, LPS treatment led to a significant increase in inflammation and oxidative stress levels, and statistical analysis showed that pretreatment with 50 μM ATR-II for 48 h reduced the LPS-induced IL-6, IL-1β, MDA and 8-OHdG levels, and increased the ratios of GSH/GSSG in the NC-shRNA MCF 10A cells, which indicated that ATR-II exerted protective effects in the LPS-induced model, while the *Nrf2* knockdown abolished the protective effects of ATR-II. We further identified the Nrf2 signaling pathway activation status through qPCR and Western blot analysis, and the results indicated that ATR-II increased the mRNA and protein levels of Nrf2 and its downstream genes *NQO1* and *HO-1*, which could contribute to the protective effect of ATR-II in LPS-induced MCF 10A cells (Figure 2H and 2I). Taken together, these results suggested that ATR-II had anti-inflammatory and antioxidant activities in the LPS-induced model, and these activities were functioned in an Nrf2/ARE-dependent manner.

### ATR-II suppressed E2-induced anchorage-independent growth of MCF 10A cells

**Figure 3:**
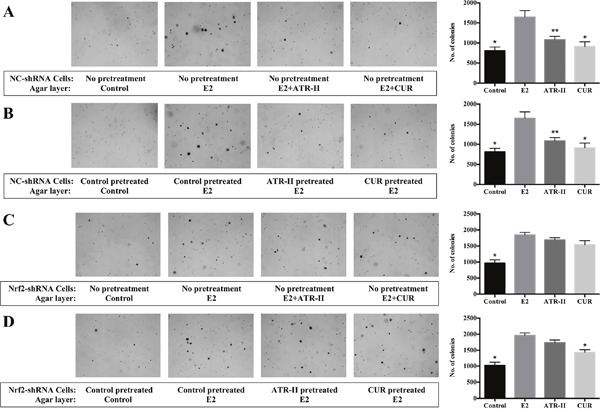
ATR-II inhibited anchorage-independent growth of MCF 10A cells, and *Nrf2* knockdown attenuated these inhibitory effects **(A)** NC-shRNA MCF 10A cells were plated in soft agar containing 0.1% DMSO (control), E2 (100 nM), E2 + ATR-II (50 μM) or E2 + CUR (20 μM) in 6-well plates for 21 days. **(B)** NC-shRNA MCF 10A cells were firstly treated with 0.1% DMSO (control), 50 μM ATR-II or 20 μM CUR 5 days, then the cells were transferred and grown in agar for additional 21 days only with presence of E2. **(C)** Nrf2-shRNA MCF 10A cells were plated in soft agar containing 0.1% DMSO (control), E2 (100 nM), E2 + ATR-II (50 μM) or E2 + CUR (20 μM) in 6-well plates for 21 days. **(D)** Nrf2-shRNA MCF 10A cells were firstly treated with 0.1% DMSO (control), 50 μM ATR-II or 20 μM CUR 5 days, then the cells were transferred and grown in agar for additional 21 days only with presence of E2. The colonies were counted under a microscope and analyzed using ImageJ software. Representative images of each group under a microscope are shown in the left panel. Graphical data are presented as the mean ± S.D. of triplicate results from three independent experiments. Significantly different (versus E2 group): *P<0.05 and **P<0.01.

The long-term exposure to estrogen could induce transformation phenotypes and genomic changes in breast epithelial cells that could develop primary breast cancers, and three mechanisms were supposed to be involved in E2 carcinogenic effects: stimulation of cellular proliferation through receptor-mediated hormonal activity, direct genotoxic effects by increasing mutation rates through a cytochrome P450-mediated metabolic activation, and induction of aneuploidy [27]. The anchorage-independent growth of transformed cells is a hallmark of carcinogenesis. To investigate the effects of ATR-II on the anchorage-independent growth of MCF 10A cells, the soft agar assay was employed. Firstly, NC-shRNA MCF 10A cells were grown in soft agar containing control (0.1% DMSO), E2 (100 nM), E2 + ATR-II (50 μM) or E2 + CUR (20 μM) for 21 days. As illustrated in Figure 3A, colony formation of MCF 10A cells was significantly reduced by ATR-II by 34.2%. The cell viability of MCF 10A cells was not affected by ATR-II treatment at concentrations of 50 μM after 3 days when examined by the MTT assay (Figure 1B). However, a continuous cell counting with trypan blue staining for 21 days revealed that the number of viable cells was significantly reduced by ATR-II at 50 μM after 10 days (data not shown). To further confirm that the inhibition of colony formation by ATR-II is not a result from cell death, NC-shRNA MCF 10A cells were pretreated with 50 μM ATR-II or 20 μM CUR for 5 days before transferred to agar. The pretreated cells were grown in agar for additional 21 days without the presence of ATR-II or CUR. As shown in Figure 3B, NC-shRNA MCF 10A cells pretreated with ATR-II and positive control CUR resulted in a significant reduced colony number by 34.4% and 35.0%, respectively. These results indicated that ATR-II inhibits the anchorage-independent growth of NC-shRNA MCF 10A cells in soft agar. Next, we investigated whether Nrf2 plays a critical role in the inhibitory effect of ATR-II in the anchorage-independent growth of MCF 10A cells, and Nrf2-shRNA MCF 10A cells were used. However, the ATR-II mediated inhibition of colony formation was remarkably reduced in Nrf2-shRNA cells (Figure 3C and 3D), and the inhibition rates were only 8.4% to 11.2%. Thus, Nrf2 played an important role in the ATR-II mediated suppression of anchorage-independent growth of MCF 10A cells.

These results suggested that ATR-II suppressed the colony formation of transformed MCF 10A cells, and Nrf2-ARE pathway is involved in the control of the anchorage-independent growth, which verified the tumor suppressive function of ATR-II *in vitro*.

### ATR-II inhibited NMU-induced mammary tumor progression in rats

**Figure 4:**
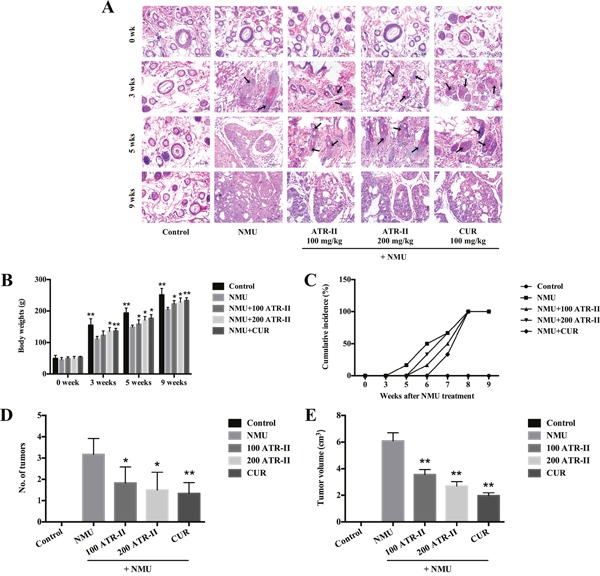
Chemopreventive effects of ATR-II on NMU-induced tumor progression in SD rats After pretreatment with control (vehicle), ATR-II (100 mg/kg and 200 mg/kg) and CUR (100 mg/kg) for 3 days, the rats were intraperitoneal injected with single dose of NMU (75 mg/kg), and then the rats were continued to treat with vehicle, ATR-II and CUR for the indicated times. **(A)** H & E staining in the mammary glands of NMU-induced rats. Mammary gland sections from each experimental group were stained with HE. Representative sections from these groups are shown (Scale bar, 50 μm). **(B)** Body weights of rats in the 7 groups during the experimental period. **(C)** Tumor incidence of palpable NMU-induced mammary tumor appearance during the experimental period. **(D)** Number of tumors at the end of experiment. **(E)** Average tumors volume at the end of experiment. Values are presented as mean ± S.D. (n=6). Significantly different (versus NMU-treated group): *P<0.05 and **P<0.01.

The previous studies indicated that ATR-II exerted chemopreventive effects *in vitro*, and we next investigate these effects *in vivo*. The NMU-induced rat at 21 days of age is a commonly accepted model for evaluation of candidate agents for chemoprevention of breast cancer [28, 29], and we explored the chemopreventive effects of ATR-II on NMU-induced mammary carcinogenesis in this study. CUR treatment was performed as positive control. Mammary tissue sections of rats were stained with Haematoxylin-eosin (H & E) staining (Figure 4A). At all four time points, the mammary glands of control group showed normal ductal morphogenesis. Ductal hyperplasia was evident as early as 3 weeks after NMU treatment, and milder hyperplasia was observed in the ATR-II and CUR treated group. At 5 weeks, the mammary tissues of NMU treated group were observed with a ductal carcinoma *in situ* (DCIS), and the increased ductal proliferation was shown in the ATR-II and CUR treated group. At 9 weeks, all the groups were observed with papillary, cribriform or mixed DCIS. As shown in Figure 4B, no significant intergroup differences were observed for the 5 groups of rats initially, however, the body weights of rats treated with NMU were significantly decreased as compared with the control group (P<0.01), and treatment with ATR-II and CUR could revert the decrease (P<0.05). The first palpable tumor in this group did not appear until 6 weeks (Figure 4C). In contrast, in the NMU-treated group, the first palpable mammary tumors appeared after 5 weeks of treatment and mammary tumor incidence was 100% after 8 weeks of NMU exposure for all the groups (Figure 4C). These rats averagely provided 3.2, 1.8, 1.5 and 1.3 of tumors to be monitored in the NMU, 100 mg/kg ATR-II, 200 mg/kg ATR-II and 100 mg/kg CUR treated groups, respectively (Figure 4D). Moreover, there was a significantly decreased mean tumor volume in the ATR-II treatment group when compared with NMU group (P<0.05, Figure 4E). These results indicated that treatment with ATR-II could inhibit the mammary tumorigenic process, revert the decrease of body weight induced by NMU, and a trend towards inhibition was observed in ATR-II groups when assessed in terms of latency, multiplicity and volume.

### ATR-II decreased inflammation and oxidative stress in NMU-induced rats

**Figure 5:**
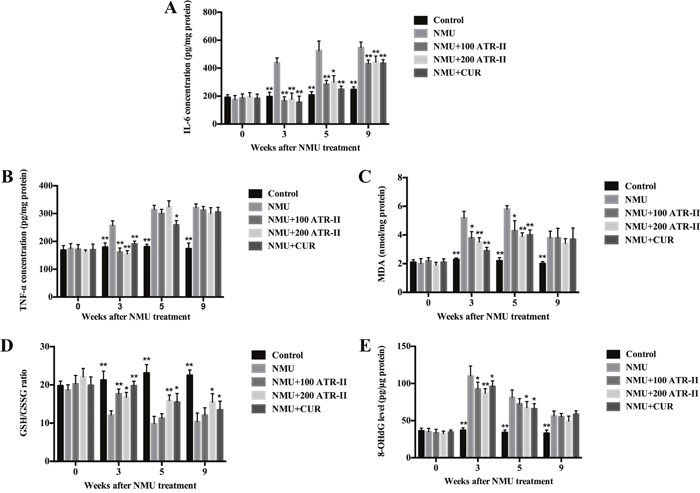
Effects of ATR-II on inflammation and oxidative stress in NMU-induced rats After pretreatment with control (vehicle), ATR-II (100 mg/kg and 200 mg/kg) and CUR (100 mg/kg) for 3 days, the rats were intraperitoneal injected with single dose of NMU (75 mg/kg), and then the rats were continued to treat with vehicle, ATR-II and CUR for the indicated times. Rat mammary tissues were analysed for IL-6 **(A)**, TNF-α **(B)**, MDA **(C)**, GSH **(D)** and 8-OHdG **(E)** levels by the commercial kits. Values are presented as mean ± S.D. (n=6). Significantly different (versus NMU-treated group): *P<0.05 and **P<0.01.

Inflammatory and oxidative injuries are closely related to the process of multi-stage carcinogenesis. To further elucidate the underlying mechanisms of inhibitory effects of ATR-II on mammary tumor progression, we measured the inflammatory and oxidative levels in rats. As shown in Figure 5, the levels of IL-6, TNF-α and 8-OHdG in rat mammary glands were detected by ELISA assay, and levels of MDA and GSH were analyzed by commercial assay kit in this study. NMU treatment increased IL-6, TNF-α, 8-OHdG and MDA levels, and decreased GSH/GSSG ratio in the mammary glands from 3 weeks of treatment. At 3 weeks, significant reduction of IL-6, TNF-α, 8-OHdG and MDA levels, and increment of GSH/GSSG ratio were observed in response to ATR-II and CUR administration. At 5 weeks, decreased IL-6, 8-OHdG and MDA levels, and increased GSH/GSSG ratio were observed (P<0.05). At 9 weeks, the level of IL-6 was significantly decreased (P<0.01), and level of GSH/GSSG ratio was significantly escalated in ATR and CUR groups when compared with NMU group (P<0.05). Thus, ATR-II abated proinflammatory cytokines (TNF-α and IL-6), reduced oxidative markers (MDA and 8-OH-dG) and increased GSH/GSSG ratio in the mammary glands, which suggested that ATR-II had anti-inflammatory and antioxidant activities in the NMU-induced rat breast cancer model.

### ATR-II increased mRNA and protein levels of Nrf2 and its downstream detoxifying enzymes in NMU-induced rats

**Figure 6:**
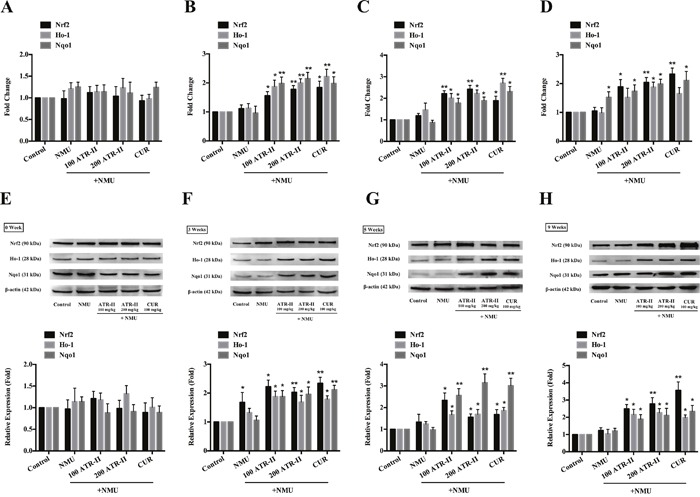
Effects of ATR-II on Nrf2, Ho-1 and Nqo1 in NMU-induced rats After pretreatment with control (vehicle), ATR-II (100 mg/kg and 200 mg/kg) and CUR (100 mg/kg) for 3 days, the rats were intraperitoneal injected with single dose of NMU (75 mg/kg), and then the rats were continued to treat with vehicle, ATR-II and CUR for the indicated times. Rat mammary tissues were collected for qPCR (**A**. 0 week, **B**. 3 weeks, **C**. 5 weeks, **D**. 9 weeks) and Western Blotting (**E**. 0 week, **F**. 3 weeks, **G**. 5 weeks, **H**. 9 weeks-tumor tissue) assay. Significantly different (versus NMU-treated group): *P<0.05 and **P<0.01.

As Nrf2 is associated with the anti-inflammatory and antioxidant effects, the induction of mRNA and protein expressions of Nrf2 and its downstream antioxidant enzymes by ATR-II in rat mammary glands were analyzed by qPCR and Western blot. As shown in Figure 6, NMU treatment alone did not change the mRNA and protein levels of Nrf2. However, administration of ATR-II and positive control CUR resulted in a significant induction of Nrf2 from 3 weeks. In addition, they induced mRNA and protein levels of the Nrf2 downstream antioxidant enzymes, Ho-1 and Nqo1. Thus, ATR-II inhibited inflammation and oxidative stress activities in the NMU-induced rats via enhancing the expression of Nrf2 and its downstream detoxifying enzymes.

### Roles of JNK and ERK signaling pathways in the ATR-II induced Nrf2-ARE activation in MCF 10A cells

**Figure 7:**
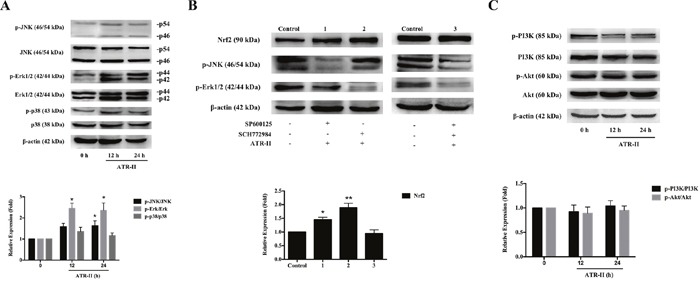
Effects of ATR-II on the phosphorylation of JNK/ERK/p38 MAPK and PI3K/Akt signaling pathways **(A)** MCF 10A cells were treated with ATR-II (50 μM) for 12 and 24 h. **(B)** Effects of SP600125 and SCH772984 on Nrf2 expression. MCF 10A cells were treated with ATR-II (50 μM) with or without the presence of SP600125 and SCH772984 for 24 h as indicated. **(C)** Effects of ATR-II on the expression and phosphorylation of PI3K and Akt. MCF 10A cells were treated with ATR-II (50 μM) for 12 and 24 h. The control group is represented by cells incubated with vehicle (0.1% DMSO). Values are presented as mean ± S.D. (n=3). Significantly different (versus control group): *P<0.05 and **P<0.01.

A number of studies have shown that several signaling pathways, including JNK/ERK/p38 MAPK [30, 31] and PI3K/Akt [32, 33] are involved in the induction of Nrf2-ARE signaling pathway. To elucidate the signal transduction pathways leading to the activation of Nrf2 and the induction of its downstream detoxifying enzymes expression in the ATR-II treated MCF 10A cells, we examined the effects of ATR-II on the expression of the phosphorylation of JNK, ERK, p38 MAPK, PI3K and Akt. Upon ATR-II treatment, time-dependent increases in the phosphorylation of JNK and Erk1/2 were observed (Figure 7A, P<0.05). To determine whether such activations of JNK and Erk1/2 contribute to the ATR-II induced Nrf2 activation, the kinase inhibitors SP600125 (for JNK) and SCH772984 (for ERK) were employed. As show in Figure 7B, inhibition of the phosphorylation of JNK and Erk1/2 did not decrease the ATR-II induced Nrf2 activation. However, SP600125 used in combination with SCH772984 significantly inhibited the ATR-II induced Nrf2 activation (Figure 7B). Moreover, no significant change was observed in the phosphorylation of PI3K or Akt on ATR-II treatment (Figure 7C, P>0.05). These results implicated the JNK and ERK, but not the p38 MAPK or PI3K/Akt, pathways were involved in ATR-II mediated *Nrf2* gene induction.

## DISCUSSION

In the present study, we investigated the induction of Nrf2-ARE signaling pathway in ATR-II treated MCF 10A cells. The results suggested that ATR-II had chemopreventive effects on E2-induced anchorage-independent growth of MCF 10A cells and NMU-induced mammary tumor progression in rats through the induction of Nrf2 and its downstream detoxifying enzymes expression via JNK/ERK-Nrf2-ARE-dependent pathways.

In traditional Chinese medicine (TCM), the rhizome of *Atractylodes macrocephala Koidz* has a long history of use for the treatment of breast cancer recurrence and metastasis, splenic asthenia, anorexia, oedema, excessive perspiration and abnormal fetal movement [34, 35]. The main active constituents in them have been proven to possess anti-tumor and anti-inflammatory activities [36]. As one of the main active constituents in the rhizome of *Atractylodes macrocephala Koidz*, atractylenolide was reported to have antioxidant and anti-inflammatory activities [16–18], indicating that it might be a potential chemopreventive agent. Based on our previous ARE-driven luciferase reporter assay, we further validated that ATR-II is an activator of the Nrf2-ARE signaling pathway, and investigated its chemopreventive effects *in vitro* and *in vivo*.

The Nrf2-ARE signaling pathway has received widespread attention in the study of cancer chemoprevention [14, 37], and Nrf2 is critically involved in the expression of the anti-inflammatory and antioxidant-associated enzymes. Under resting condition, the negative regulatory protein Kelch-like ECH2 related protein 1 (KEAP1) could capture Nrf2 in the cytoplasm and function as an adaptor for Cul3-based E3 ligase to promote proteasomal degradation of Nrf2; under stress condition, Nrf2 could decouple from KEAP1 and transfer into cell nucleus, then binds to antioxidant responsive element (ARE) and induces the expression of cytoprotective protein (HO-1, NQO1, GST, GCLC, SRXN1 and TXNRD1, etc.) by clearing ROS and increased cell resistance to oxidative stress. Besides, several protein kinase pathways are involved in the activation of Nrf2 (such as MAPK [38, 39], PI3K [40, 41] and PKC [42]), and Nrf2 could be cap-independently translated [43]. Furthermore, epigenetic regulation is also an important mechanism in Nrf2 activation. Several studies have shown that many dietary phytochemicals could induce Nrf2-ARE pathway via regulating histone deacetylase (HDACs) and DNA methyltransferase (DNMTs) [26, 44]. Our results indicated that induction of JNK and ERK pathways might be important mechanisms in the activation of Nrf2-ARE signaling pathway, and other mechanism such as epigenetic regulation remains to be investigated in the next study.

Activation of the Nrf2 pathway is a promising strategy for chemoprevention, however, some investigation has revealed that Nrf2 and its downstream detoxifying enzymes are overexpressed in various cancer cell lines and human cancer tissues, giving cancer cells an advantage for survival and growth [45, 46]. In addition, Nrf2 is upregulated in multiple drug resistant (MDR) cancer cells, which accounts for the acquired chemoresistance [47, 48]. The E2 induced MCF 10A cell model indicated that ATR-II had chemopreventive effects on MCF 10A cells associated with activating the Nrf2-ARE signaling pathway. However, ATR-II could decrease the proinflammatory cytokines and oxidative markers levels at early time points (3 and 5 weeks, the palpable tumors did not appear), while the TNF-α, MDA and 8-OHdG levels did not change at 9 weeks (after the palpable tumors appeared) treatment in NMU induced rats. Thus it might be necessary to activate the Nrf2 pathway while chemoprevention, and inhibit it during chemotherapy.

Inflammation and oxidative stress are considered to be risk factors of breast cancer all along, and studies have shown that chronic inflammation could intensify DNA damages, oncogene mutations and genomic instability in normal cells, and excess ROS could cause oxidative damages to macromolecular substances in the body (such as DNA, proteins and unsaturated fatty acids, etc.), thus promoting the occurrence and development of tumorigenesis [22, 49]. IL-6 and TNF-α are two proinflammatory cytokines that well recognized to have close relationship with promotion and progression of cancer. GSH is capable of preventing damage to important cellular components caused by reactive oxygen species. MDA results from lipid peroxidation of polyunsaturated fatty acids, and the degree of lipid peroxidation could be estimated by the amount of MDA. 8-OHdG is an oxidized derivative of deoxyguanosine, being a frequent product of DNA oxidation and a mutagenic lesion in DNA. During DNA replication, unrepaired 8-OHdG lesions induce G-T transversions, which may contribute to carcinogenesis [50]. In our study, we validated the anti-inflammatory and antioxidant capacity of ATR-II by determining these proinflammatory cytokines and oxidative markers, which is significant for its chemopreventive effects.

**Figure 8:**
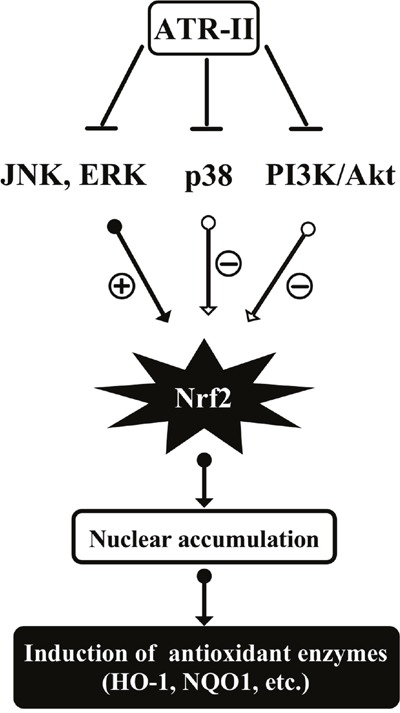
Proposed mechanisms of atractylenolide II (ATR-II) induced activation of Nrf2 and subsequent expression of antioxidant enzymes in MCF10A cells ATR-II could activate JNK or Erk1/2 phosphorylation, which in turn phosphorylates Nrf2. This will facilitate the nuclear translocation of Nrf2 and induce subsequent expression of antioxidant enzymes in MCF10A cells.

In conclusion, ATR-II activated JNK and ERK signaling pathways and facilitated the release of Nrf2 for nuclear translocation, thus inducing the expression of antioxidant enzymes (HO-1 and NQO1) in MCF 10A cells. The proposed mechanisms of ATR-II induced activation of Nrf2 and subsequent expression of antioxidant enzymes are shown in Figure 8, and these results add further evidence of the molecular mechanisms that allow ATR-II to exert protective effects and its potential role as a chemopreventive agent in mammary carcinogenesis.

## MATERIALS AND METHODS

### Reagents

ATR-II and curcumin (CUR) were purchased from Chengdu Must Biological Technology Co., Ltd. (Chengdu, China). MTT and N-Nitroso-N-methylurea (NMU) were purchased from Sigma Chemical Co. Ltd. (St. Louis, MO, USA). 17β-Estradiol (E2) was purchased from Cayman Chemical Co. (Ann Arbor, MI). Primary antibodies against HO-1, PI3 Kinase p85, phospho-PI3 Kinase p85 (Tyr458) (p-PI3K), Akt, phospho-Akt (Ser 473) (p-Akt) were obtained from Cell Signaling Technology (Beverly, MA, USA), primary antibody against Nrf2 was from Abcam Inc. (Cambridge, UK), primary antibody β-actin was from TransGen Biotech. (Beijing, China), primary antibody against NQO1 was from ImmunoWay Biotechnology (Newark, USA), and primary antibody against p38, phospho-p38 (p-p38), JNK, phospho-JNK (p-JNK), Erk1/2 and phospho-Erk1/2 (p-Erk) were from Wanleibio (Shenyang, China). ELISA kit for 8-hydroxydeoxyguanosine (8-OHdG) was purchased from Uscn Life Science Inc. (Wuhan, China), ELISA kits for rat IL-6, rat TNF-α, human IL-6 and human IL-1β were from NeoBioscience Technology Co., Ltd. (Shanghai, China), Glutathione (GSH) and glutathione disulfide (GSSG) assay kit and lipid peroxidation malondialdehyde (MDA) assay kit from Beyotime Biotechnology (Haimen, China).

### Cell culture, cell viability assay and lentiviral transduction

The human mammary epithelial MCF 10A cell line was obtained from the Chinese Academy of Sciences Cell Bank (Shanghai, China), and was cultured as described previously [51] in a 1:1 mixture of Dulbecco's modified Eagle and Ham F12 (DMEM/F12; Gibco, USA) medium supplemented with 5% heat-inactivated horse serum (Gibco, USA), 10 μg/ml insulin, 100 ng/ml Cholera toxin, 0.5 μg/ml hydrocortisone, 20 ng/ml recombinant EGF, 2 mM L-glutamine, 100 μg/ml penicillin/streptomycin mixture at 37 °C in a 5% CO_2_ atmosphere.

The effects of ATR-II and CUR on the viability of MCF 10A cells were detected by MTT assay. MCF 10A cells were seeded in 96-well plates at an initial density of 8000 cells/well for 24 h. The cells were then treated with ATR (1-1000 μM) and CUR (1-200 μM) for 24, 48 and 72 h. Then an MTT assay was performed as reported [52].

Lentivirus mediated short hairpin RNAs were used to establish stable mock (NC-shRNA) and Nrf2 knockdown (Nrf2-shRNA) MCF 10A cells. The shRNA clone sets were obtained from Genecopoeia (Rockville, MD, USA), and lentiviral transduction was performed according to the manufacturer's manual. After selection in DMEM/F12 medium supplemented with geneticin (G418) for 3 weeks, the NC-shRNA and Nrf2-shRNA cells were further used to evaluate the functional role of Nrf2.

### Animal and experimental procedures

Experimental procedures were carried out in accordance with the Guide for the Care and Use of Laboratory Animals, and before the animal experiments were carried out, the procedures were approved by the Committee of Scientific Research and the Committee of Animal Care of the West China Hospital, Sichuan University (Chengdu, China). Female Sprague-Dawley (SD) rats were purchased from Experimental Animal Center of the Dashuo Biotechnology (Chengdu, China). At 21 days of age, the rats were intraperitoneal injection with control (solvent) or 75 mg/kg NMU. Rats receiving control (solvent) were fed with vehicle, while those receiving NMU were fed with vehicle, ATR-II or CUR 3 days before and after intraperitoneal injection of NMU. Six rats were sacrificed at time points of 3, 5 and 9 weeks following injection for each group. Mammary glands were collected and stored at −80 °C or fixed in formalin for further analysis.

### Protein lysate preparation and western blot analysis

Protein of MCF 10A cells and rat mammary tissues were isolated by lysis buffer (Beyotime, China), and the concentrations were measured using bicinchoninic acid (BCA) (Beyotime, China). The protein samples were then separated on 10% SDS-PAGE and transferred onto the PVDF membranes (Bio-Rad, CA). After blocked with 5% BSA (Roche, USA) in TBST for 1 h, the cropped membranes were incubated with primary antibodies overnight at 4 °C. Membranes were washed three times and incubated with the corresponding peroxidase-conjugated secondary antibodies for 1 h at 37 °C. Membranes were washed again with TBST, and then were scanned with Bio-Rad Gel Doc XR documentation system and band density was determined using Image Lab 5.0 software (Bio-Rad, Hercules, CA, USA).

### RNA isolation and quantitative PCR analysis

RNA was extracted from MCF 10A cells and rat mammary tissue using RNAiso plus reagent (Takara, Japan), and the extracted RNA was reverse transcribed using the RevertAid™ First Strand cDNA Synthesis Kit (Thermo Scientific, USA). For quantitative PCR analysis, Bestar^®^ SybrGreen qPCR Mastermix (DBI^®^ Bioscience, Germany) was used according to the manufacturer's protocol. Sequences of PCR primers were designed by Primer Express v3.0 (Applied Biosystems, USA) and were verified with BLAST in GenBank database. The sequences of the primers are shown in Table 1. The quantitative PCR was performed using an iQ5 Real Time PCR system (Bio-Rad, USA). Average gene C_T_ values were normalized to the average GAPDH C_T_ values of the same cDNA sample.

**Table 1:** Primers of human and rat Nrf2, HO-1, NQO1 and GAPDH for quantitative PCR analysis

Oligo Name	Source	Sequence (5′-3′)
Nrf2-F	Human	5′-CCTCAACTATAGCGATGCTGAATCT-3′
Nrf2-R	Human	5′-AGGAGTTGGGCATGAGTGAGTAG-3′
HO-1-F	Human	5′-TCCGATGGGTCCTTACACTC-3′
HO-1-R	Human	5′-TAAGGAAGCCAGCCAAGAGA-3′
NQO1-F	Human	5′-GGTTTGAGCGAGTGTTCATAGG-3′
NQO1-R	Human	5′-GGTTTGAGCGAGTGTTCATAGG-3′
GAPDH-F	Human	5′-GCCTCAAGATCATCAGCAATGC-3′
GAPDH-R	Human	5′-CCTTCCACGATACCAAAGTTGTCAT-3′
Nrf2-F	Rat	5′-CTCTCTGAACTCCTG- GACGG-3′
Nrf2-R	Rat	5′-GGGTCTCCGTAAATGGAAG-3′
HO-1-F	Rat	5′-GCTCTATCGTGCTCGCATGA-3′
HO-1-R	Rat	5′-AATTCCCACTGCCACGGTC-3′
NQO1-F	Rat	5′-TCACAGGGGAGCCGAAGGACT-3′
NQO1-R	Rat	5′-GGGGTGTGGCCAATGCTGTA-3′
GAPDH-F	Rat	5′-GGTGCTGAGTATGTCGTGGAG-3′
GAPDH-R	Rat	5′-CAGTCTTCTGAGTTGGCAGTGATG-3′

### ELISA and MDA, GSH/GSSG assay

For measurement of MDA, GSH/GSSG, IL-6, TNF-α, IL-1β and 8-OHdG, the supernatants of cell culture medium, cell lysis or rat mammary tissue lysis were collected, and then the assays were performed according to the manufacturer's protocol.

### Cell immunofluorescence analysis

To determine the expression of Nrf2 in MCF 10A cells after treatment with ATR-II and CUR, immunohistofluorescence (IF) was performed on the cells. MCF 10A cells were fixed with 4% paraformaldehyde for 10 min, and then permeabilized with 0.1% Triton X-100 (Amresco, USA) solution for 10 min at 4 °C. After over 3 h of blocking in 5% BSA, the primary antibody against Nrf2 was added to the blocking solution at a dilution of 1:100, and incubated overnight at 4 °C. Subsequently, cells were incubated with FITC tagged secondary antibody (ZSGB-BIO, China) for 1 h at 37 °C, and then 1mg/ml of DAPI (Sigma, USA) was used to stain the nucleus. Finally, cells were viewed with an epifluorescence microscope (Nikon 80i, Japan).

### Colony formation assay

The NC-shRNA and Nrf2-shRNA MCF 10A cells (1.5 × 10^5^/well) were seeded in 1.5 ml of DMEM/F12 containing 0.3% agar over 3 ml of DMEM/F12 containing 0.6% agar with 5% horse serum in 6-well plates. The cells were maintained with control (0.1% DMSO), E2, E2 + ATR-II (20 μM), E2 + ATR-II (50 μM) and E2 + CUR (20 μM) at 37 °C in a humidified 5% CO_2_ atmosphere for 21 days.

In another set of experiment, the MCF 10A cells were first treated with control (0.1% DMSO), ATR-II at 20 and 50 μM or CUR at 20 μM for 5 days. On day 5, the pretreated MCF 10A cells (1.5 × 10^5^/well) were seeded in 1.5 ml of DMEM/F12 containing 0.3% agar over 3 ml of DMEM/F12 containing 0.6% agar with 5% horse serum in 6-well plates. The cells were maintained in soft agar without the presence of ATR-II or CUR at 37 °C in a humidified 5% CO_2_ atmosphere for additional 21 days.

The colonies were photographed using a computerized microscope system with the Nikon 80i and counted based on the usage of ImageJ.

### Statistical analysis

The data are presented as the mean ± S.D. (standard deviation). The statistical analysis was performed using one-way analysis of variance (ANOVA). P values less than 0.05 were considered statistically significant.
